# PDGF-induced PI3K-mediated signaling enhances the TGF-β-induced osteogenic differentiation of human mesenchymal stem cells in a TGF-β-activated MEK-dependent manner

**DOI:** 10.3892/ijmm.2013.1606

**Published:** 2013-12-27

**Authors:** JUN YOKOTA, NAOYUKI CHOSA, SHUNSUKE SAWADA, NAOTO OKUBO, NORIKO TAKAHASHI, TOMOKAZU HASEGAWA, HISATOMO KONDO, AKIRA ISHISAKI

**Affiliations:** 1 Division of Cellular Biosignal Sciences, Department of Biochemistry, Iwate Medical University, Yahaba, Iwate 028-3694, Japan; 2 Department of Prosthodontics and Oral Implantology, Iwate Medical University School of Dentistry, Morioka, Iwate 020-8505, Japan; 3 Division of Periodontology, Department of Conservative Dentistry, Iwate Medical University School of Dentistry, Morioka, Iwate 020-8505, Japan; 4 Clinical Research Laboratory, Iwate Medical University School of Dentistry, Morioka, Iwate 020-8505, Japan; 5 Department of Pathophysiology and Therapeutics, Division of Pharmasciences, Faculty of Pharmaceutical Sciences, Hokkaido University, Sapporo 060-0812, Japan; 6 Department of Pediatric Dentistry, Tokushima University Hospital, Tokushima 770-8504, Japan

**Keywords:** transforming growth factor-β, platelet-derived growth factor, osteogenic differentiation, proliferation, mesenchymal stem cells, extracellular signal-regulated kinase, phosphoinositide-3-kinase

## Abstract

Transforming growth factor-β (TGF-β) is a critical regulator of osteogenic differentiation and the platelet-derived growth factor (PDGF) is a chemoattractant or mitogen of osteogenic mesenchymal cells. However, the combined effects of these regulators on the osteogenic differentiation of mesenchymal cells remains unknown. In this study, we investigated the effects of TGF-β and/or PDGF on the osteogenic differentiation of human mesenchymal stem cells (hMSCs). The TGF-β-induced osteogenic differentiation of UE7T-13 cells, a bone marrow-derived hMSC line, was markedly enhanced by PDGF, although PDGF alone did not induce differentiation. TGF-β induced extracellular signal-regulated kinase (ERK) phosphorylation and PDGF induced Akt phosphorylation. In addition, the mitogen-activated protein kinase (MAPK)/ERK kinase (MEK) inhibitor, U0126, suppressed the osteogenic differentiation induced by TGF-β alone. Moreover, U0126 completely suppressed the osteogenic differentiation synergistically induced by TGF-β and PDGF, whereas the phosphoinositide-3-kinase (PI3K) inhibitor, LY294002, only partially suppressed this effect. These results suggest that the enhancement of TGF-β-induced osteogenic differentiation by PDGF-induced PI3K/Akt-mediated signaling depends on TGF-β-induced MEK activity. Thus, PDGF positively modulates the TGF-β-induced osteogenic differentiation of hMSCs through synergistic crosstalk between MEK- and PI3K/Akt-mediated signaling.

## Introduction

Bone formation and remodeling occur throughout development and adult life. The formation of new bone is a complex cascade, involving cell proliferation, osteogenic cell differentiation, extracellular matrix (ECM) maturation and matrix mineralization. Bone remodeling depends on osteoblasts (OBs), osteocytes and osteoclasts (1). Mesenchymal stem cells (MSCs) differentiate into OBs and synthesize and secrete bone matrix, which subsequently becomes mineralized tissue. Once embedded into the bone matrix, OBs further differentiate into osteocytes.

MSCs were first derived from bone marrow and are characterized by their self-renewal ability and their capacity to develop into a variety of mesenchymal tissues (2–4). The expansion of human bone marrow-derived MSCs (BM-MSCs) *in vitro* and their subsequent autoimplantation may be used for stem cell therapy without the risk of rejection by the immune system. BM-MSCs differentiate into OBs, chondrocytes and adipocytes (5) and are therefore considered the main source of bone regeneration and remodeling during homeostasis (6–9). Much of this process depends on the ability of MSCs to proliferate and differentiate under the influence of biologically active molecules (i.e., growth factors) (10–13). The role of growth factors in bone repair is widely recognized, particularly for platelet-derived growth factor (PDGF), insulin-like growth factor-I (IGF-I), vascular endothelial growth factor (VEGF) and transforming growth factor-β (TGF-β), all of which are inducers, particularly in osteoprogenitor cells (14). These growth factors are usually stored in the ECM; however, following injury, they are actively released by the ECM, cells and platelets.

TGF-β is one of the most abundant growth factors in the bone matrix (15) and regulates osteoblastic differentiation in a variety of ways, such as by stimulating the proliferation and development of early OBs, although it inhibits their maturation and mineralization (16). TGF-β is released from the bone surface and recruits MSCs to bone-resorptive sites, where they undergo differentiation into mature OBs, thus coupling bone resorption with bone formation (17). TGF-β activates intracellular effectors, such as mitogen-activated protein kinases (MAPKs) and Sma- and Mad-related proteins (Smads) (18–20). There are at least three distinctly regulated groups of MAPKs: extracellular signal-related kinases (ERKs), Jun N-terminal kinases (JNKs) and p38 MAPKs (p38). The activation of the ERK pathway mediates the differentiation of BM-MSCs and that of the pre-adipocyte cell line, 3T3 L1, into mature adipocytes. It also regulates the proliferation and differentiation of bone cells and BM-MSCs during osteogenic differentiation (21). JNK and p38 are activated in human and mouse OBs to regulate bone resorption (22,23).

PDGF is a polypeptide growth factor secreted from cytokine-laden granules of aggregated platelets early after tissue injury (24,25). PDGF is mainly produced by platelets and has been implicated in the repair of tissue damage, such as fractures (26). PDGF consists of A, B, C and D isoforms, and forms homo or hetero dimers, such as PDGF-AA or PDGF-AB (26). PDGF-BB exhibits the strongest activity of these isoforms (26) and has been approved by the US Food and Drug Administration (FDA) for the treatment of patients with bone defects in oral and maxillofacial regions (27–30). However, the specific molecular mechanisms by which PDGF regulates the activity of multiple cell types to control tissue development are not yet fully understood. Much of the research in this area has focused on the role of PDGF in controlling the vascularization of nascent tissue, forming within the wound site (31). PDGF indirectly regulates bone regeneration by increasing the expression of angiogenic molecules, such as VEGF (32), hepatocyte growth factor (33) and that of the proinflammatory cytokine, interleukin-6 (34); VEGF is a particularly important molecule in bone regeneration (35). In general, PDGF binding leads to autophosphorylation on multiple tyrosine residues, thereby activating several downstream cascades, such as ERK belonging to MAPKs, phosphoinositide-3-kinase (PI3K)/Akt, Janus kinase (JAK) and signal transducer and activator of transcription (STAT) pathways (36,37). Osteogenic progenitor cells respond to PDGF ligand-binding by the activation of Src tyrosine kinases (38–40) and of the Akt protein kinase and Grb2-mediated ERK-signaling (40). Consequently, PDGF increases the pool of osteogenic cells at the injury site, acting as a chemotactic agent and mitogen (41).

Even though the effects of TGF-β or PDGF alone on the osteogenic differentiation of undifferentiated mesenchymal cells have been reported in detail (17,40,42), their combined effects still remain unknown to date. In this study, we investigated the osteogenic differentiation of human MSCs (hMSCs) following stimulation with exogenous TGF-β and PDGF. We also investigated the mechanisms through which intracellular signals induced by TGF-β and/or PDGF control the osteogenic differentiation of hMSCs.

## Materials and methods

### Reagents

Recombinant human TGF-β and PDGF, as well as the MAPK/ERK kinase (MEK) inhibitor, U0126, and the PI3K inhibitor, LY294002, were purchased from Calbiochem (La Jolla, CA, USA).

### Cell culture and osteogenic differentiation

The human BM-MSC line, UE7T-13, the lifespan of which was prolonged by infection with a retrovirus encoding human papillomavirus E7 and human telomerase reverse transcriptase (hTERT) (43,44), was purchased from the Health Science Research Resources Bank (JCRB no. 1154, Japan Health Sciences Foundation, Tokyo, Japan). The UE7T-13 cells were cultured in Dulbecco’s modified Eagle’s medium (DMEM; Sigma, St. Louis, MO, USA) supplemented with 10% fetal bovine serum (FBS; PAA Laboratories, Piscataway, NJ, USA) at 37°C in a humidified incubator with an atmosphere of 5% CO_2_. To induce osteogenic differentiation, the UE7T-13 cells were cultured in 24-well culture plates (Nunc, Roskilde, Denmark) containing basal osteogenic differentiation medium (BODM) [α-MEM (Sigma) supplemented with 100 nM dexamethasone (Sigma), 50 μg/ml ascorbic acid (Nacalai Tesque, Kyoto, Japan), 10 mM β-glycerophosphate (Sigma) and 10% FBS (PAA Laboratories)] containing TGF-β and/or PDGF. Half of the medium in each dish was changed every 2–3 days.

### Alkaline phosphatase (ALP) staining

The UE7T-13 cells were cultured in 24-well plastic culture plates or Osteologic™ discs (BD Biosciences, Franklin Lakes, NJ, USA) (a proprietary hydroxyapatite substitute for bone mineral) containing BODM supplemented with TGF-β and/or PDGF for 1 week. The surface of the Osteologic cell culture disc is coated with calcium phosphate. The cells were then stained with ALP using the TRAP/ALP staining kit (Wako Pure Chemical Industries, Ltd., Osaka, Japan) according to the manufacturer’s instructions.

### Alizarin red staining

Confluent UE7T-13 cells were cultured in 24-well plastic culture plates containing BODM supplemented with TGF-β and/or PDGF. After 2 weeks, bone matrix mineralization was evaluated by Alizarin red S (Sigma) staining. Alizarin red was extracted by the addition of 10% cetylpyridinium chloride (Sigma) in 8 mM Na_2_HPO_4_ (Wako Pure Chemical) and 1.5 mM KH_2_PO_4_ (Wako Pure Chemical Industries, Ltd.) while the absorbance was measured on an MPR-A4i microplate reader (Tosoh Co., Tokyo, Japan) at 540 nm.

### RNA isolation and quantitative RT-PCR (qRT-PCR)

Confluent UE7T-13 cells in 24-well plastic culture plates or Osteologic discs were cultured in BODM containing TGF-β and/or PDGF. After 1 week of culture, total RNA was isolated using Isogen reagent (Nippon Gene Co., Ltd., Tokyo, Japan) according to the manufacturer’s instructions. cDNA was synthesized from total RNA with the PrimeScript RT reagent kit (Takara Bio, Inc., Shiga, Japan). qRT-PCR was performed on a Thermal Cycler Dice Real Time System (Takara Bio, Inc.) with SYBR Premix Ex Taq II (Takara Bio, Inc.) and specific oligonucleotide primers (presented in Table I). The mRNA expression levels of runt-related transcription factor 2 (RUNX2), ALP, liver/bone/kidney (ALPL), collagen, type I, alpha 1 (COL1A), secreted phosphoprotein 1 (osteopontin, SPP1), integrin-binding sialoprotein (bone sialoprotein, IBSP), and bone gamma-carboxyglutamate (gla) protein (osteocalcin, BGLAP) were normalized to glyceraldehyde-3-phosphate dehydrogenase (GAPDH), and the relative expression levels were expressed as the fold change relative to the corresponding control.

### Western blot analysis

The UE7T-13 cells were washed twice with PBS and then lysed in RIPA buffer (50 mM Tris-HCl, pH 7.2, 150 mM NaCl, 1% NP-40, 0.5% sodium deoxycholate, and 0.1% SDS) containing protease and phosphatase inhibitor cocktails (Sigma). The protein content was measured with BCA reagent (Pierce Biotechnology, Inc., Rockford, IL, USA). Equivalent protein samples were separated by 10–20% SDS-polyacrylamide gradient gel electrophoresis (SDS-PAGE) and transferred onto a polyvinylidene difluoride (PVDF) membrane (Millipore, Billerica, MA, USA). After blocking with 5% non-fat dry milk in TTBS (50 mM Tris-HCl, pH 7.2, 150 mM NaCl, and 0.1% Tween-20), the membrane was incubated with a primary anti-Akt (Cell Signaling Technology, Inc., Danvers, MA, USA), anti-phospho-Akt (Ser473) (p-Akt, Cell Signaling Technology, Inc.), anti-p44/42 MAPK (ERK1/2, Cell Signaling Technology, Inc.), anti-phospho-p44/42 MAPK (Thr202/Tyr204) (p-ERK, Cell Signaling Technology, Inc.) antibodies, and anti-β-actin (clone C4, Santa Cruz Biotechnology, Santa Cruz, CA, USA) antibody as the loading control for normalization. The blots were then incubated with ALP-conjugated secondary antibody and developed using the BCIP/NBT membrane phosphatase substrate system (KPL). Densitometry was performed using ImageJ software (version 1.44). Data are expressed as the ratio of phosphorylated to total molecular bands.

### Statistical analysis

Data are presented as the means ± standard deviation (SD). Statistical analysis was performed by using the Student’s t-test, and values of p<0.05 were considered to indicate statistically significant differences.

## Results

### PDGF markedly enhances the TGF-β-induced ECM mineralization in hMSCs

**Figure 1:**
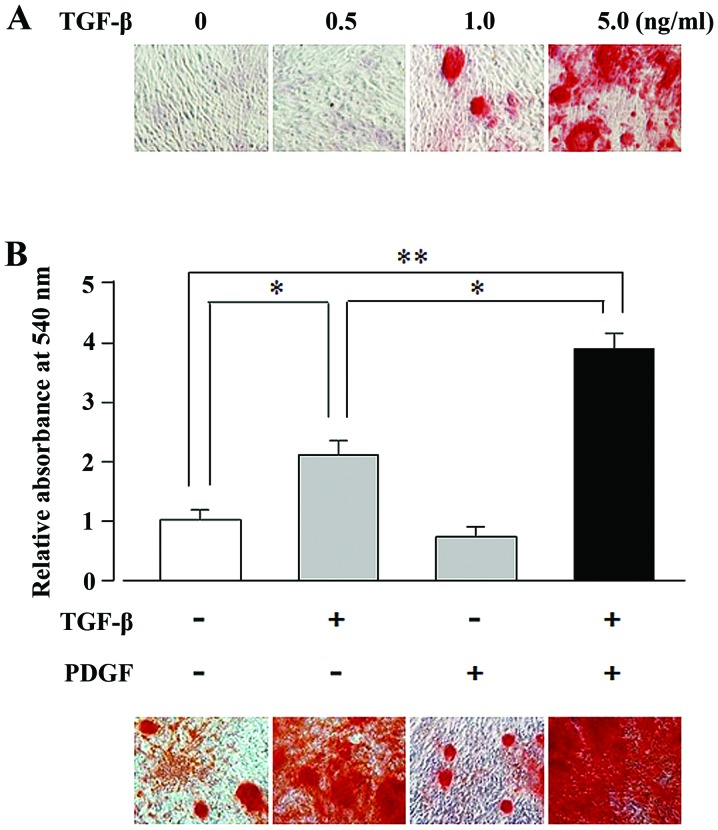
Platelet-derived growth factor (PDGF) markedly enhanced the transforming growth factor-β (TGF-β)-induced extracellular matrix (ECM) mineralization in human mesenchymal stem cells (hMSCs); PDGF alone had no effect on cells cultured on plastic culture plates. (A) UE7T-13 cells were cultured in plastic culture plates containing basal osteogenic differentiation medium (BODM) supplemented with various concentrations of TGF-β (0.5–5.0 ng/ml). Two weeks later they were stained with Alizarin red. (B) UE7T-13 cells were cultured in BODM containing 5.0 ng/ml TGF-β and/or 10.0 ng/ml PDGF. After 2 weeks, the bone matrix mineralization of the cells was assessed by Alizarin red staining (lower panel). Alizarin red was extracted with 10% cetylpyridinium chloride and absorbance was measured at 550 nm (upper panel). Data are presented as the means ± SD. ^*^p<0.05, ^**^p<0.02 indicate statistifcal significance.

In general, mesenchymal cells that differentiate into OBs induce the mineralization of the the ECM (45–47). We investigated the TGF-β-mediated induction of the osteogenic differentiation of the BM-MSC cell line, UE7T-13, by using Alizarin red staining to assess ECM mineralization. As illustrated in Fig. 1A, TGF-β induced matrix mineralization in the UE7T-13 cells in a dose-dependent manner (1.0–5.0 ng/ml). We then examined the synergistic effects of PDGF and TGF-β on osteogenic differentiation. Alizarin red staining of TGF-β- and PDGF-stimulated cells revealed that the TGF-β (5 ng/ml)-induced matrix mineralization was enhanced by PDGF (10 ng/ml), whereas PDGF alone did not induce mineralization (Fig. 1B).

### TGF-β and PDGF synergistically upregulate the transcript levels of ALPL and IBSP in hMSCs

**Figure 2:**
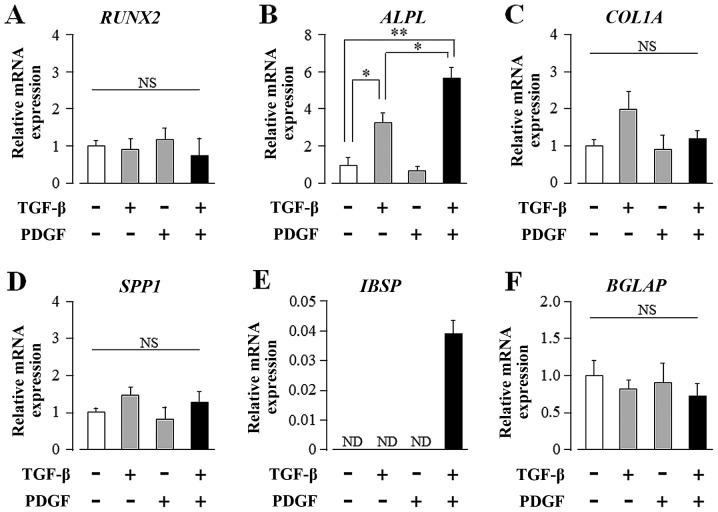
Transforming growth factor-β (TGF-β) and platelet-derived growth factor (PDGF) synergistically upregulate the transcript expression of alkaline phosphatase (ALP), liver/bone/kidney (ALPL) and integrin-binding sialoprotein (bone sialoprotein, IBSP) in human mesenchymal stem cells (hMSCs) cultured on plastic culture plates. Confluent UE7T-13 cells were cultured on 24-well plastic culture plates with basal osteogenic differentiation medium (BODM) containing 5.0 ng/ml TGF-β and/or 10.0 ng/ml PDGF. After 1 week culture, qRT-PCR was performed with specific oligonucleotide primers (presented in Table I). mRNA expression levels of (A) runt-related transcription factor 2 (RUNX2), (B) ALPL, (C) collagen, type I, alpha 1 (COL1A), (D) secreted phosphoprotein 1 (osteopontin, SPP1), (E) IBSP, and (F) bone gamma-carboxyglutamate (gla) protein (osteocalcin, BGLAP) were normalized to GAPDH, and the results are expressed as the fold change relative to the respective control. Data are presented as the means ± SD. ^*^p<0.05, ^**^p<0.02 indicate statistical significance.

To elucidate the molecular mechanisms underlying the synergistic effects of TGF-β and PDGF on osteogenic differentiation, we investigated the transcript expression of osteogenic differentiation markers in UE7T-13 cells by qRT-PCR. As shown in Fig. 2B, TGF-β alone markedly induced ALPL mRNA expression; PDGF alone had no effect on ALPL expression. Intriguingly, PDGF markedly enhanced the TGF-β-induced upregulation of ALP mRNA expression. In addition, TGF-β and PDGF greatly induced IBSP expression, although neither regulator alone was sufficient to influence IBSP expression (Fig. 2E). These regulators, either alone or synergistically, had no effect on the transcript expression of other osteogenic differentiation marker genes, such as RUNX2, COL1A1, SPP1 and BGLAP (Fig. 2A, C, D and F, respectively).

### PDGF enhances the TGF-β-induced osteogenic differentiation of hMSCs on a proprietary hydroxyapatite substitute for bone mineral (Osteologic discs)

**Figure 3:**
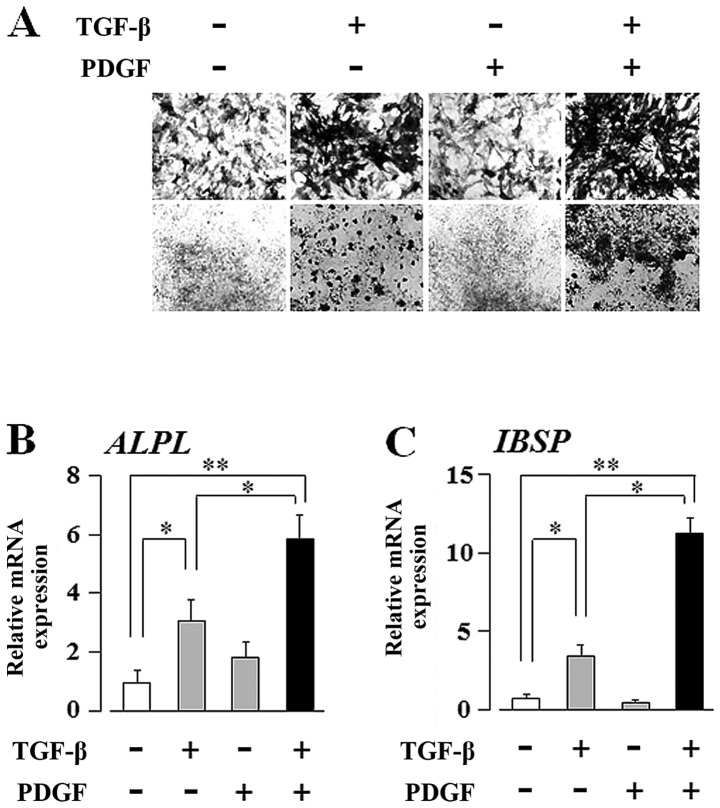
Platelet-derived growth factor (PDGF) enhances the transforming growth factor-β (TGF-β)-induced osteogenic differentiation of human mesenchymal stem cells (hMSCs) on a proprietary hydroxyapatite substitute for bone mineral (Osteologic discs); PDGF alone had no effect. UE7T-13 cells were cultured in basal osteogenic differentiation medium (BODM) containing 5.0 ng/ml TGF-β and/or 10.0 ng/ml PDGF on Osteologic discs. After 1 week of culture, (A) alkaline phosphatase (ALP) and (B) ALP, liver/bone/kidney (ALPL) and (C) integrin-binding sialoprotein (bone sialoprotein, IBSP) mRNA expression levels were assessed. (B and C) data are presented as the means ± SD. ^*^p<0.05, ^**^p<0.02 indicate statistical significance.

As shown in Figs. 1 and 2B, PDGF enhanced the TGF-β-induced osteogenic differentiation of hMSCs, whereas PDGF alone had no effect on differentiation. These results suggest TGF-β is superior to PDGF in the hierarchy that mediates the osteogenic differentiation of hMSCs. However, as shown in Fig. 2E, it was unclear which growth factor is the main regulator of IBSP mRNA expression. In order to clearly rank these growth factors in the context of promoting OB differentiation, we examined the mechanisms through which TGF-β and/or PDGF affect the differentiation of hMSCs on a proprietary hydroxyapatite substitute for bone mineral (Osteologic discs), instead of the plastic culture plates utilized in Figs. 1 and 2. The surface of the Osteologic cell culture disc is coated with calcium phosphate as described in Materials and methods. As shown in Fig. 3A, ALP staining of the UE7T-13 cells cultured on Osteologic discs revealed that PDGF clearly enhanced the TGF-β-induced upregulation of ALP expression, whereas PDGF alone did not. In addition, as shown in Fig. 3B and C, qRT-PCR revealed that PDGF clearly enhanced the TGF-β-induced upregulation of ALPL and IBSP transcript expression, whereas PDGF alone did not. Thus, the Osteologic culture system clearly demonstrated that TGF-β is superior to PDGF in the regulation of the osteogenic differentiation of hMSCs.

### TGF-β upregulates the PDGF-induced Akt activity, whereas PDGF downregulates the TGF-β-induced ERK activity

**Figure 4:**
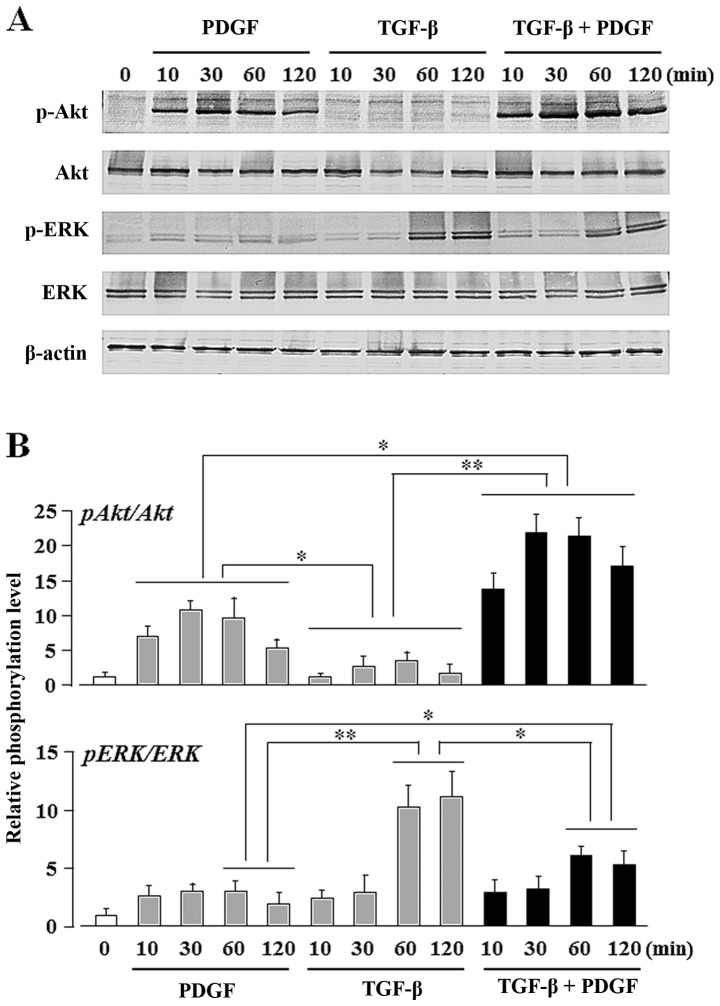
Transforming growth factor-β (TGF-β) activates extracellular signal-regulated kinase (ERK) but not Akt; platelet-derived growth factor (PDGF) activates Akt but not ERK. UE7T-13 cells cultured in 24-well plastic culture plates containing growth medium were serum-starved overnight, then stimulated with 5.0 ng/ml TGF-β and/or 10.0 ng/ml PDGF. (A) Phosphorylation status was analyzed by western blot analysis. (B) Densitometry was performed with ImageJ software. Data are expressed as the ratio of the phosphorylated to total molecular bands. Data are presented as the mean ± SD. ^*^p<0.05, ^**^p<0.02 indicate statistical significance.

In order to identify the signaling pathways activated by TGF-β and/or PDGF during the osteogenic differentiation of UE7T-13 cells, we evaluated the phosphorylation status of the PI3K/Akt- and ERK-mediated pathways. Gharibi *et al* previously reported that the PDGF BB-induced crosstalk between these pathways affects the proliferation and adipogenic commitment of hMSCs: PDGF-BB-induced PI3K/Akt signaling enhanced the proliferative activity of the hMSCs, and PDGF-BB-induced ERK-mediated signaling suppressed the adipogenic differentiation of hMSCs (48). In general, cell proliferation is poorly compatible with differentiation and proliferation/differentiation switches have been demonstrated in different cell types (49–51). In addition, a reciprocal correlation exists between the adipogenetic and osteogenetic differentiation of undifferentiated mesenchymal cells (52–54). Thus, the crosstalk between the PI3K/Akt- and ERK-mediated pathways appears to affect the osteogenic commitment of hMSCs. In our study, ERK phosphorylation was upregulated by stimulation with TGF-β alone, but Akt phosphorylation was unaffected (Fig. 4). By contrast, Akt phosphorylation was upregulated by stimulation with PDGF alone, but ERK phosphorylation was not. The phosphorylation of both ERK and Akt was detected after co-stimulation of PDGF and TGF-β. Notably, the combined stimulation of TGF-β and PDGF strongly induced Akt phosphorylation (Fig. 4B; bar graph on upper panel, lanes 10–13), whereas PDGF alone only moderate induced Akt phosphorylation (Fig. 4B; bar graph on upper panel, lanes 2–5). By contrast, TGF-β alone markedly induced ERK phosphorylation (Fig 4B; bar graph on lower panel, lanes 8 and 9), whereas the combination of TGF-β and PDGF moderately induced ERK phosphorylation (Fig 4B; bar graph on lower panel, lanes 12 and 13). No phosphorylation of Smad2/3 was detected, although it is one of the major pathways of TGF-β stimulation in these cells (data not shown).

### PDGF-induced PI3K-mediated signaling enhances the TGF-β-induced, MEK-dependent osteogenic differentiation of hMSCs

**Figure 5:**
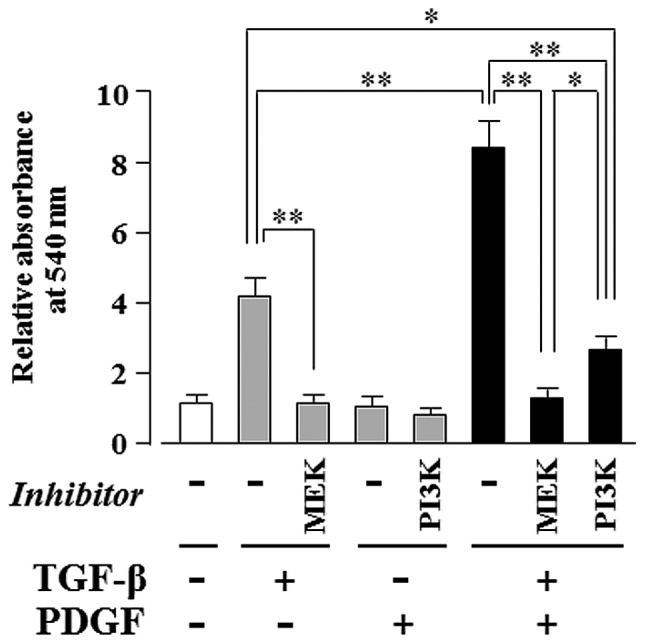
Platelet-derived growth factor (PDGF)-induced PI3K-mediated signaling enhances transforming growth factor-β (TGF-β)-induced osteogenic differentiation of human mesenchymal stem cells (hMSCs) in a TGF-β-activated MEK-dependent manner. UE7T13 cells were treated with 5.0 ng/ml TGF-β and/or 10.0 ng/ml PDGF for 2 weeks and ECM mineralization was assessed by Alizarin red staining. The MEK inhibitor, U0126 (10.0 μM), and the PI3K inhibitor, LY294002 (2.0 μM), were added to the culture medium 30 min prior to the addition of TGF-β and/or PDGF. Data are presented as the means ± SD. ^*^p<0.05, ^**^p<0.02 indicate statistical significance.

As shown in Fig. 5, TGF-β alone markedly induced ECM mineralization in a MEK-dependent manner (Fig. 5; lanes 2 and 3); moreover, PDGF clearly enhanced the TGF-β-induced ECM mineralization (Fig. 5; lane 6), whereas PDGF alone had no effect (Fig. 5; lane 4). The synergistically induced ECM mineralization was completely suppressed by U0126 (a MEK inhibitor) (Fig. 5; lane 7) and only partially suppressed by LY294002 (a PI3K inhibitor) (Fig. 5; lane 8). The level of ECM mineralization was lower in the culture supplemented with TGF-β, PDGF and LY294002 (Fig. 5; lane 8) than in the culture supplemented with TGF-β alone (Fig. 5; lane 2).

## Discussion

TGF-β is crucial for connective tissue regeneration and bone remodeling, as demonstrated by several *in vivo* and *in vitro* studies. It affects osteogenic differentiation and bone formation (55–59) and increases the mRNA levels of osteogenic differentiation markers and ALP activity in murine bone marrow MSCs (57). In this study, we investigated whether TGF-β promotes the osteogenic differentiation of the human bone marrow-derived MSC line, UE7T-13. Our results demonstrated that TGF-β induced the osteogenic differentiation of UE7T-13 in a dose-dependent manner (1.0–5.0 ng/ml) (Fig. 1A). Thus, we focused on the synergistic effects of multiple growth factors on the osteogenic differentiation of MSCs.

PDGF is mainly produced by platelets and has tissue repair functions, such as fracture repair (26). In addition, PDGF is a chemoattractant or mitogen of osteogenic mesenchymal cells (41), and does not seem to directly affect the osteogenic differentiation of MSCs. As shown in Fig. 1B, PDGF alone did not induce ECM mineralization in the UE7T-13 cells; however, PDGF markedly enhanced the TGF-β-induced ECM mineralization.

We also investigated the synergistic effects of TGF-β and PDGF on the mRNA expression of osteogenic differentiation marker genes. qRT-PCR revealed that the transcript expression of ALPL, a mineralization-associated enzyme, was increased by stimulation with TGF-β alone (Fig. 2B). PDGF markedly enhanced the TGF-β induction of ALPL, whereas PDGF alone had no effect. The TGF-β-induced ALPL expression was similarly and greatly increased by PDGF stimulation, whereas PDGF alone had no effect (data not shown). It is now well established that osteogenic differentiation is marked by sequential stages of cellular proliferation and ECM maturation (60). ALPL expression is a transient early marker of osteogenic differentiation in MSCs, peaking at the end of the proliferative stage before ECM maturation (61). Therefore, our findings suggest that the PDGF support of TGF-β-induced osteogenic differentiation may be important during the early stages of the osteogenesis of MSCs.

IBSP expression is restricted to mineralized connective tissues (62). IBSP is a phosphorylated, sulfated glycoprotein that represents one of the major non-collagenous ECM proteins associated with mineralized tissues (63–65). A high expression of IBSP coincides with *de novo* bone formation (62). IBSP is primarily expressed by mature OBs and osteoclasts, as well as by hypertrophic chondrocytes (66). We previously reported that OB-like SaOS-2 cells have an increased expression of IBSP on titanium surfaces coated with hydroxyapatite (67). Thus, the expression of IBSP is a useful indicator of osteogenic differentiation. IBSP expression occurs at the middle-to-late-stages of osteogenic differentiation of undifferentiated mesenchymal cells (68). In our study, IBSP mRNA expression was detected only in cultures containing both TGF-β and PDGF, but was not detected in the cultures containing TGF-β or PDGF alone (Fig. 2E). The bone surface is comprised of hydroxyapatite, a calcium phosphate mineral, on which MSCs differentiate into OBs; this of course differs significantly from a polystyrene culture dish surface. Therefore, we examined the osteogenic response of hMSCs to TGF-β and/or PDGF in cultures grown on Osteologic discs, a proprietary hydroxyapatite substitute. As shown in Fig. 3C, TGF-β alone markedly induced IBSP mRNA expression in the UE7T-13 cells cultured on Osteologic culture discs. In addition, PDGF clearly enhanced the TGF-β-induced IBSP mRNA expression, whereas PDGF alone had no effect on IBSP in this culture system. ALP staining and qRT-PCR revealed that PDGF clearly enhanced the TGF-β-induced upregulation of ALP protein and ALPL mRNA expression; PDGF alone had no effect (Fig. 3A and B). Thus, the Osteologic culture system demonstrated that TGF-β is superior to PDGF in the osteogenic differentiation of hMSCs. The supportive effect of PDGF seems to occur during the early stage (for ALPL induction) to late stage (for IBSP induction) of osteogenic differentiation.

In order to clarify the intracellular signaling pathways that mediate the interaction between PDGF and TGF-β in the induction of the osteogenic differentiation of MSCs, we evaluated the phosphorylation status of the PI3K/Akt and ERK pathways. The MEK inhibitor, U0126, completely suppressed the TGF-β-induced ECM mineralization in the UE7T-13 cell culture (Fig. 5; lane 3); PDGF alone did not promote osteogenic activity (Fig. 5; lane 4), but enhanced the TGF-β-induced ECM mineralization (Fig. 5; lane 6). This synergistic promotion of ECM mineralization was completely suppressed by U0126 (Fig. 5; lane 7), strongly suggesting that PDGF enhances the TGF-β-induced osteogenic differentiation of hMSCs in a TGF-β-activated MEK-dependent manner. In addition, the synergistic differentiation of hMSCs by both factors was partially suppressed by the PI3K inhibitor, LY294002 (Fig. 5; lane 8). Taken together, our results indicate that PDGF-induced PI3K-mediated signaling enhances the TGF-β-induced osteogenic differentiation of hMSCs in a TGF-β-activated MEK-dependent manner. Notably, the level of ECM mineralization in the presence of TGF-β, PDGF and LY294002 (Fig. 5; lane 8) was markedly lower than that in the cultures with TGF-β alone (Fig. 5; lane 2). As described above, PDGF inhibits the TGF-β-induced MEK activity (Fig. 4B: bar graph on lower panel, lanes 12 and 13), whereas TGF-β enhances PDGF-induced Akt activity (Fig. 4B: bar graph on upper panel, lanes 10–13). These results suggest that ECM mineralization may be predominantly induced by PI3K/Akt-mediated signaling than by MEK/ERK-mediated signaling in the presence of both factors.

Thus, it can be concluded that PDGF-stimulated PI3K/Akt-mediated signaling enhances the TGF-β-induced osteogenic differentiation of hMSCs in a MEK/ERK-dependent manner. The combination of PDGF-activated PI3K/Akt and TGF-β-activated MEK mediates osteogenic differentiation which is important for optimizing the potential therapeutic use of hMSCs for bone formation. Our findings provide insight into the establishment of novel therapeutic methods for bone formation by hMSCs.

## Figures and Tables

**Table I:** Primer sequences.

Full name	Symbol	Primer sequence (5′→3′)	
Runt-related transcription factor 2	*RUNX2*	Forward	CACTGGCTGCAACAAGA
		Reverse	CATTCCGGAGCTCAGCAGAATAA
Alkaline phosphatase, liver/bone/kidney	*ALPL*	Forward	GGACCATTCCCACGTCTTCAC
		Reverse	CCTTGTAGCCAGGCCCATTG
Collagen, type I, alpha 1	*COL1A*	Forward	TCTAGACATGTTCAGCTTTGTGGAC
		Reverse	TCTGTACGCAGGTGATTGGTG
Secreted phosphoprotein 1	*SPP1*	Forward	ACACATATGATGGCCGAGGTGA
		Reverse	TGTGAGGTGATGTCCTCGTCTGTAG
Integrin-binding sialoprotein	*IBSP*	Forward	GGCCACGATATTATCTTTACAAGCA
		Reverse	TCAGCCTCAGAGTCTTCATCTTCA
Bone gamma-carboxyglutamate (gla) protein	*BGLAP*	Forward	AGGTGCAGCCTTTGTGTCCA
		Reverse	GGCTCCCAGCCATTGATACAG
Glyceraldehyde-3-phosphate dehydrogenase	*GAPDH*	Forward	GCACCGTCAAGGCTGAGAAC
		Reverse	ATGGTGGTGAAGACGCCAGT
